# Measuring types and timing of childhood maltreatment: The psychometric properties of the KERF-40+

**DOI:** 10.1371/journal.pone.0273931

**Published:** 2022-09-08

**Authors:** Katja I. Seitz, Sarah Gerhardt, Claudius von Schroeder, Angelika Panizza, Dorothea Thekkumthala, Katja Bertsch, Sabine C. Herpertz, Christian Schmahl, Inga Schalinski

**Affiliations:** 1 Department of General Psychiatry, Center for Psychosocial Medicine, Medical Faculty, Heidelberg University, Heidelberg, Germany; 2 Department of Addictive Behavior and Addiction Medicine, Central Institute of Mental Health, Medical Faculty Mannheim, University of Heidelberg, Mannheim, Germany; 3 Department of Psychosomatic Medicine and Psychotherapy, Central Institute of Mental Health, Mannheim, Germany; 4 Department of Psychology, University of Konstanz, Konstanz, Germany; 5 Department of Psychology, Ludwig-Maximilians-University Munich, Munich, Germany; 6 Psychologische Hochschule Berlin, Berlin, Germany; Harvard Medical School, UNITED STATES

## Abstract

Childhood maltreatment, specifically during sensitive developmental periods, is a major risk factor for poor physical and mental health. Despite its enormous clinical relevance, there is still a lack of scales measuring different types, timing, and duration of childhood maltreatment. The current study sought to validate and determine the psychometric properties of the brief German version of the Maltreatment and Abuse Chronology of Exposure (MACE) scale, the KERF-40. The KERF-40 was administered as an interview (i.e., KERF-40-I) to 287 adult participants with and without mental disorders. Based on item response theory, items of the KERF-40-I were assigned to different types of maltreatment, resulting in a scaled version, the KERF-40+. Test-retest reliability was assessed in a small subsample (*n* = 14). Convergent and relative predictive validity were measured with correlations of the KERF-40+ and the Childhood Trauma Questionnaire (CTQ) as well as self-report measures of general and trauma-related psychopathology. Rasch analysis and fit statistics yielded a 49-item version, encompassing ten different types of maltreatment. The test-retest reliability of the KERF-40+ was shown to be acceptable to excellent for almost all global and subscale scores (.74 ≤ ρ ≤ 1.00), with the exception of the subscale emotional neglect (ρ = .55). Convergent validity with the CTQ was confirmed for both KERF-40+ global scores (.72 ≤ *r* ≤ .87) and corresponding subscale scores (.56 ≤ *r* ≤ .78). Relative predictive validity was reflected by significant small-to-moderate correlations between KERF-40+ global scores and indices of general and trauma-related psychopathology (.24 ≤ *r* ≤ .45). Taken together, the KERF-40+ appears to be suited for clinicians and researchers interested in retrospectively assessing different types, timing, and duration of childhood maltreatment experiences during sensitive periods in adults.

## Introduction

Affecting more than half of all children worldwide, childhood maltreatment represents a major public concern [1], particularly because of its lifelong negative consequences for physical and mental health [e.g., 2]. Childhood maltreatment is commonly defined as any act of abuse or neglect by a parent or other caregiver, which leads to harm, potential for harm, or threat of harm to a child [3]. Broader definitions of childhood maltreatment additionally include further potentially harmful experiences before the age of 18 years, such as peer victimization and witnessing domestic violence [1]. Despite the high prevalence and lifelong consequences, diagnostic instruments which reliably assess different types, timing, and duration of childhood maltreatment are still lacking. Such instruments are urgently needed for deepening our understanding of childhood maltreatment-related health outcomes and underlying pathophysiological mechanisms.

In large-scale studies, exposure to childhood maltreatment has been reported to exert pervasive effects on physical and mental health throughout the life course [2, 4]. Specifically, childhood maltreatment has been identified as a risk factor for cancer, cardiovascular, respiratory and sexually transmitted diseases as well as various mental disorders [2, 4, 5]. Consequently, substantial annual costs have been attributed to childhood maltreatment and its associated health outcomes with an estimated US $581 billion in Europe and $748 billion in North America [6]. Considering the enormous individual and economic burden related to childhood maltreatment, it appears crucial for research, clinical practice, and public health to reliably and comprehensively assess such adverse experiences in order to identify those in need of therapeutic interventions. Currently, the most commonly used scales to assess childhood maltreatment include the Childhood Trauma Questionnaire [CTQ; 7] and Adverse Childhood Experiences (ACE) scale [4]. However, these scales have considerable limitations [8, 9]. First, like most of the existing scales, the CTQ and ACE adopt a narrow definition of childhood maltreatment, focusing exclusively on emotional, physical and sexual abuse, as well as emotional and physical neglect by primary caregivers. Thus, other important types such as bullying by peers, witnessing violence toward siblings or parents, or physical and emotional abuse by siblings cannot be captured with those scales. Bullying by peers has widely been recognized as an important risk factor for poor mental health outcomes, including depression, anxiety, non-suicidal self-injury, and suicidal ideation [10–12]. Witnessing violence toward siblings has been characterized as a potent but understudied type of childhood maltreatment, being associated with elevated symptom levels of depression, anxiety, somatization, anger/aggression, and dissociative symptoms [13, 14]. Abuse by siblings has also recently been identified as a relevant factor for different mental health symptoms [13, 15], however, abuse by siblings is one of the least researched and recognized types of domestic violence [16, 17]. Thus, these findings underscore the need to address bullying by peers, witnessing violence toward siblings and parents, and abuse by siblings in addition to commonly measured types of childhood abuse and neglect when aiming for a comprehensive assessment of childhood maltreatment. Second, most of the existing scales do not consider the timing and duration of childhood maltreatment exposure. In line with the notion of stress-sensitive developmental periods, recent studies suggest that type, timing, and duration of childhood maltreatment may be influential for later mental health outcomes, interpersonal functioning, and neurobiological measures [18–21]. Hence, type, timing, and duration should be assessed to gain a comprehensive understanding of the risk pathways leading from childhood maltreatment to psychopathology. Third, although self-report questionnaires are less costly and time-consuming than interviews, higher agreement between retrospective interviews rather than questionnaires with prospective measures of childhood maltreatment may support employing interviews [22], specifically when aiming for an in-depth assessment of childhood maltreatment experiences.

Taking these limitations into account, researchers developed the Maltreatment and Abuse Chronology of Exposure (MACE) scale [9]. The MACE has been administered as an interview as well as a self-report questionnaire. The original experimental version of the MACE, the MACE-X, encompasses 75 questions to evaluate timing, duration, and severity of retrospectively reported exposure to ten types of maltreatment during each year of childhood up to the age of 18 years in adults. Besides commonly assessed types of childhood maltreatment (i.e., emotional, physical and sexual abuse as well as emotional and physical neglect), the MACE covers additional types, such as peer verbal abuse, peer physical bullying, witnessing interparental violence, and witnessing violence toward siblings. The US [9], German [23], and Norwegian [8] versions of the MACE show good to excellent psychometric properties, making it a recommendable choice in the retrospective assessment of childhood maltreatment in adults [24]. Moreover, the global measures of the US and Norwegian versions of the MACE have been shown to account for substantially more variance in mental health symptoms than the CTQ and ACE [8, 9]. So far, the MACE has already been successfully employed in several studies targeting different sequelae of childhood maltreatment, such as alterations in brain structure, function, and connectivity [25–32], aberrant neurocognitive functioning [33, 34], deviant endocrine regulation as indicated by hair cortisol concentration [35], sleep disruptions [36], and different forms of psychopathology [37–40].

As some circumstances (e.g., research projects with large study samples) may require less time-consuming measures than the original German interview version of the MACE, the KERF ["Belastende Kindheitserfahrungen"; 23], Isele and colleagues developed a brief German interview version of the MACE, the so-called KERF-40-I. To date, the KERF-40-I has not been validated and its psychometric properties have not been determined yet. Thus, the overarching aim of the present study was to validate the KERF-40-I and to determine its psychometric properties, leading to the development of the KERF-40+. Specifically, the study aims were to (1) select the most suitable items from the KERF-40-I based on item response theory to measure ten different types of childhood maltreatment, (2) determine the test-retest reliability of the KERF-40+, (3) measure the convergent validity between the KERF-40+ and the CTQ [7], and (4) evaluate the relative predictive validity of the KERF-40+ as compared to the CTQ with regard to measures of general psychopathology [i.e., Brief Symptom Inventory, BSI; 41], depressive symptomatology [i.e., revised version of Beck Depression Inventory, BDI-II; 42], posttraumatic stress disorder (PTSD) symptom severity [i.e, PTSD Checklist for DSM-5, PCL-5; 43], and dissociative symptoms [i.e., German adaptation of the Dissociative Experience Scale, the Fragebogen zu dissoziativen Symptomen, FDS; 44].

## Materials and methods

### Setting and ethics

The present study was part of the German Research Foundation’s Research Training Group 2350, dedicated to investigating the impact of adverse childhood experiences on psychosocial and somatic conditions across the lifespan [45]. Qualified diagnosticians (i.e., with at least a master’s degree in clinical psychology) administered the brief version of the KERF as an interview, the KERF-40-I. Additionally, diagnosticians conducted the German version of the Structured Clinical Interview for DSM-5 [SCID-5; 46] to assess current and past mental disorders. All diagnosticians received standardized diagnostic training before the beginning of the study.

The present study was approved by the ethics committees of the medical faculties of Heidelberg University in Heidelberg and Mannheim, Germany. All participants provided written informed consent prior to participation and were reimbursed for their participation.

### Participants

Two hundred and eighty-seven individuals completed the KERF-40-I, including 206 participants with mental disorders and 81 participants without a current or past mental disorder (i.e., healthy volunteers). Participants with mental disorders were recruited via clinical referral from inpatient and outpatient units and advertisements, while healthy volunteers were recruited via advertisements. An important objective of our recruitment procedure was to ensure that both individuals with mental disorders and healthy volunteers covered a broad range of childhood maltreatment experiences. Thus, in our study advertisements, we described our goal of investigating the consequences of childhood maltreatment experiences in adults with and without mental disorders, specifically to target those individuals who have had such experiences. In addition, in most of the Research Training Group’s projects, the German version of the Childhood Trauma Screener [CTS; 47] was used as part of the telephone screening process to assess childhood maltreatment experiences of potential participants. By using the CTS, we were able to ensure that particularly individuals without a current or past mental disorder (i.e., healthy volunteers), who are not known for being at risk of having a history of childhood abuse or neglect, also reported different types and intensities of childhood maltreatment experiences. Following inclusion, the self-report CTQ [48] was used to ensure that our sample covered a broad range of childhood maltreatment experiences.

Inclusion criteria for all participants comprised being between 18 and 60 years of age at the time of recruitment and German language fluency. Exclusion criteria for all participants were neurological disorders, severe medical illness, lifetime diagnoses of schizophrenia, schizoaffective, or bipolar I disorder, and pregnancy. Further exclusion criteria for healthy volunteers were current or past mental disorders. Demographic and clinical characteristics of the study sample are summarized in Table 1.

**Table 1:** Sample characteristics.

Characteristic^a^	Total sample (*N* = 287)	Individuals with mental disorders (*n* = 206)	Healthy volunteers (*n* = 81)
Demographic information			
Age in years	33.05 (11.48)	33.21 (11.67)	32.64 (11.03)
Female, No. (%)	205 (71.4)	148 (71.8)	57 (70.4)
School education in years	12.19 (1.44)	12.05 (1.53)	12.56 (1.14)
Childhood trauma			
CTQ sum score^b^	50.52 (20.17)	54.10 (20.33)	41.34 (16.65)
CTQ Emotional Abuse^b^	12.89 (6.40)	14.12 (6.30)	9.71 (5.52)
CTQ Physical Abuse^b^	8.04 (4.43)	8.38 (4.54)	7.14 (4.01)
CTQ Sexual Abuse^b^	7.52 (4.74)	7.91 (5.01)	6.52 (3.84)
CTQ Emotional Neglect^b^	13.93 (6.05)	15.12 (5.91)	10.87 (5.30)
CTQ Physical Neglect^b^	8.16 (3.56)	8.58 (3.70)	7.10 (2.93)
Clinical symptom severity			
BSI GSI^b^	0.90 (0.62)	1.06 (0.58)	0.33 (0.37)
BDI-II total score^b^	15.78 (13.82)	21.79 (13.06)	4.03 (4.74)
PCL-5 total score^b^	23.92 (18.41)	27.59 (18.11)	10.52 (12.39)
FDS DES score^b^	14.95 (11.90)	16.83 (12.04)	8.21 (8.54)
Current mental disorder, No. (%)			
Affective Disorders	82 (28.6)	82 (39.8)	0 (0)
PTSD	53 (18.5)	53 (25.7)	0 (0)
Anxiety and Obsessive Compulsive Disorders	43 (15.0)	43 (20.9)	0 (0)
Somatic symptom and related disorders	41 (14.3)	41 (19.9)	0 (0)
Eating Disorders	8 (2.8)	8 (3.9)	0 (0)
Substance Use Disorders	27 (9.4)	27 (13.1)	0 (0)

BDI-II = Beck Depression Inventory revised; BSI GSI = Brief Symptom Inventory Global Severity Index; CTQ = Childhood Trauma Questionnaire; FDS = German adaptation of the Dissociative Experience Scale, the Fragebogen für dissoziative Symptome; PCL-5 = PTSD Checklist for DSM-5; PTSD = posttraumatic stress disorder.

^a^ Data are presented as mean (*SD*) unless otherwise indicated.

^b^ A subsample of the present sample provided data on the CTQ (*N* = 281), BSI (*N* = 224), BDI-II (*N* = 195), PCL-5 (*N* = 223) and FDS (*N* = 218).

### Measures

#### KERF-40+

The original German version of the MACE, the KERF [23], includes 75 questions measuring exposure to ten types of childhood maltreatment up to the age of 18 years: Parental Verbal Abuse (PEA), Parental Non-Verbal Emotional Abuse (PNVEA), Parental Physical Abuse (PPA), Sexual Abuse (SEXA), Emotional Neglect (EN), Physical Neglect (PN), Peer Verbal Abuse (PEERE), Peer Physical Bullying (PEERP), Witnessed Interparental Violence (WITP), and Witnessed Violence Toward Siblings (WITS). When a question targets childhood maltreatment experiences which involve one of two different groups (i.e., parents or siblings, and peers or a romantic partner, respectively), one question may encompass two items (i.e., version A and B of the same question). For example, item A may target a particular childhood maltreatment experience involving parents or peers, while item B may target the same maltreatment experience involving siblings or a romantic partner. By asking the same question about one particular childhood maltreatment experience involving, for example, parents and/or siblings, the KERF allows for a swift assessment of childhood maltreatment experiences involving different perpetrator groups. Items are answered dichotomously (i.e., yes or no) according to whether the interviewee experienced this type of childhood maltreatment in the first 18 years of his or her life, and for each year of exposure. Eight items address positive experiences (e.g., family as a source of strength and support), hence answers to these items are reversed. For the recoded items, the overall item response is defined as at least one year of exposure (i.e., lacking the positive experience).

The brief interview version of the original KERF, the KERF-40-I, encompasses 40 questions assessing exposure to ten types of childhood maltreatment before the age of 18 years: Parental Emotional Abuse (PEA), Parental Physical Abuse (PPA), Sexual Abuse (SEXA), Emotional Neglect (EN), Physical Neglect (PN), Physical and Emotional Abuse by Peers (PEER), Witnessed Violence Towards Parents (WITP), Witnessed Violence Toward Siblings (WITS), Emotional Abuse by Siblings (SEA), and Physical Abuse by Siblings (SPA). Altogether, 14 out of 40 questions were repeated in respect to different potential perpetrator groups (i.e., either parents and/or siblings or peers and/or someone in a romantic relationship). The additional information resulted in 54 items that were available for scaling. In comparison to the original version of the KERF, the KERF-40-I also considers the exposure to sibling violence.

In the process of validating the KERF-40-I, we again revised the existing ten subscales, leading to the development of the KERF-40+. Please refer to the S1 Table for similarities and differences between the KERF-40-I and the KERF-40+. According to previously described procedures [e.g., 8], we calculated the following KERF-40+ scores to use in our statistical analyses:

- Ten subscale scores, reflecting the severity of all items with positive responses for each subscale. Each subscale is fitted into a 0 to 10-point scale.
- A KERF-40+ sum score, reflecting the sum of the scores on the ten subscales and ranging between 0 and 100.
- A KERF-40+ multiplicity score, reflecting the number of different types of maltreatment (i.e., according to the KERF-40+ subscale definition) and ranging between 0 and 10. For the KERF-40+ multiplicity score, each type of maltreatment is required to be reported above a cutoff level (see Method section, Convergent validity).
- A KERF-40+ duration score, reflecting the number of years with exposure to at least one type of maltreatment above a cutoff level. The KERF-40+ duration score ranges between 0 and 18 years.

#### Childhood trauma questionnaire (CTQ)

The German version of the CTQ [48] was used to determine the convergent validity of the KERF-40+. The CTQ is a 28-item self-report questionnaire measuring emotional, physical, and sexual abuse as well as emotional and physical neglect [7]. The five subscales targeting childhood maltreatment consist of five items each. Items are rated on a five-point Likert scale ranging from 1 (*not at all*) to 5 (*very often*). Scores on the total scale range from 25 to 125, and subscale scores range from 5 to 25, with higher scores indicating more severe abuse or neglect. Cutoff scores of 8 (Sexual Abuse) 10 (Physical Abuse, Physical Neglect), 13 (Emotional Abuse), and 15 (Emotional Neglect) have been proposed to indicate the presence of moderate to severe childhood maltreatment [49]. In a subsample of the present sample which provided data on the CTQ (*N* = 281), the internal consistency of four of the five subscales ranged from good (i.e., Cronbach’s α = .85 for Physical Abuse) to excellent (i.e., Cronbach’s α = .94 for Sexual Abuse); Cronbach’s alpha for the Physical Neglect subscale was at .68.

#### Indices of general and trauma-specific psychopathology

The German version of the BSI [41], the German version of the BDI-II [50], the German version of the PCL-5 [43], and the German adaptation of the Dissociative Experience Scale, the FDS [44] were used to evaluate the relative predictive validity of the KERF-40+ as compared to the CTQ with regard to generic and trauma-specific measures of psychopathology.

*Brief Symptom Inventory (BSI)*. The BSI [41], a 53-item self-report questionnaire, was used to assess past-week clinically relevant symptoms in nine dimensions (i.e., Somatization, Obsession-Compulsion, Interpersonal Sensitivity, Depression, Anxiety, Hostility, Phobic Anxiety, Paranoid Ideation, and Psychoticism). Items are scored on a 5-point Likert scale ranging from 0 (*not at all*) to 4 (*extremely*). The BSI Global Severity Index (BSI GSI), defined as the mean of all items, ranges from 0 to 4, with higher scores reflecting more severe general psychopathology. A BSI GSI cutoff score of 0.62 has been suggested to indicate significant psychological distress [41]. In a subsample of the present sample which provided data on the BSI (*N* = 224), the BSI demonstrated excellent internal consistency, Cronbach’s α = .96.

*Beck Depression Inventory*, *revised version (BDI-II)*. The BDI-II [50], a 21-item self-report questionnaire, was administered to assess depressive symptoms in the preceding two weeks with 21 items on a four-point Likert-scale. Scores on the total scale range from 0 to 63, with higher scores reflecting more severe depressive symptoms. Criteria have been proposed to interpret the total score as reflecting mild (14–19), moderate (20–28), or severe (29–63) depression [42]. In a subsample of the present sample which provided data on the BDI-II (*N* = 195), the BDI-II demonstrated excellent internal consistency, Cronbach’s α = .96.

*PTSD Checklist for DSM-5 (PCL-5)*. The PCL-5 [43], a 20-item self-report questionnaire, was used to measure PTSD symptom severity in the past-month. Items are rated on a 5-point Likert scale ranging from 0 (*not at all*) to 4 (*extremely*). Total scores range from 0 to 80, with higher scores indicating more severe PTSD symptomatology. Cutoff scores between 28 and 37 have been proposed to indicate a probable PTSD diagnosis [51, 52]. In a subsample of the present sample which provided data on the PCL-5 (*N* = 223), the PCL-5 demonstrated excellent internal consistency, Cronbach’s α = .94.

*German adaptation of the Dissociative Experiences Scale (DES)*, *the Fragebogen für dissoziative Symptome (FDS)*. The FDS [44], a 44-item self-report questionnaire, was administered to measure how frequently different dissociative experiences such as dissociative amnesia, absorption, depersonalization, derealization, and conversion occur. Items are scored on a scale from 0 (*never*) to 100 (*always*). The FDS DES score is the mean value from all frequency estimates of the 28 original DES items. In a subsample of the present sample which provided data on the FDS (*N* = 218), the FDS demonstrated excellent internal consistency, Cronbach’s α = .92.

### Statistical analysis

Analyses were performed in R version 4.0.2 [53] and SPSS version 27 (IBM SPSS Statistics for Windows, Armonk, NY, USA: IBM Corp.).

#### Item to subscale assignment and scaling

Initially, we included all items of the KERF-40-I that were available for inclusion for each childhood maltreatment subtype. The analysis was based on the content of the items and considered similar evaluation guidelines as in the original version of the KERF [23]. First, we used the scoring protocol that referred to ten different subscales (see S1 Table). For those subscales that could not be fitted in a Rasch model, alternative item assignment was considered based on the content.

We determined a simple Rasch model for each subscale, aiming to include at least four items that measure the latent trait [see 9]. If we failed to identify a simple Rasch model according to the initial evaluation proposal, we considered revised versions of the initial subscale until we determined an adequate fit considering our quality criteria described below. The different items of a subscale will have varying levels of item difficulty, which will reflect the latent trait dimension. Taking the subscale Parental Physical Abuse (PPA) as an example, individuals are expected to report the item “intentionally pushed, grabbed, shoved, slapped, pinched, punched or kicked you” (item 6A) more often than specific types of physical maltreatment such as “spanked you with an object such as a strap, belt, brush, paddle, rod, etc.” (item 8A), and only individuals with the highest levels of latent exposure are expected to report the item “hit you so hard that you were physically injured” (item 9A). Therefore, we expected individuals with lower levels of exposure to report items with a lower severity, while we expected those individuals with higher levels of latent exposure levels to endorse easier as well as more severe items of the corresponding subscale. In order to detect Rasch-based models for each subscales and design figures (see S1 Fig), we used R version 4.0.2 [53] and the packages “extended Rasch Modelling” [eRM; 54] and “latent trait models” [Itm; 55]. First, the mean square fit criteria (*X*^2^/df) as measured by infit and outfit mean square fits, were evaluated to select appropriate items. While the infit parameter captures unpredicted reports on the item with a similar difficulty to the individual exposure levels, the outfit parameter depicts unpredicted reports on items that are different from the individual exposure level. There is a general recommendation that acceptable mean square fits should not exceed values of 1.5, and should not be lower than 0.5. However, there are also more strict criteria of a maximum of 1.3 and a minimum of 0.7 for mean square fits [56]. For the current study, we aimed to identify fits falling into the range of 0.7 to 1.3. Items with fits between 1.3 and 1.5 or 0.5 and 0.7 were critically checked prior to their inclusion, and only considered for inclusion in order to meet the minimum number of items per subscale or for content purposes (i.e., balance the number of items for potential perpetrator groups). Furthermore, outfits were checked before infits. Higher mean square fits indicated randomness in the data and were therefore treated stricter compared to lower mean square fits (i.e., showing item responses that are too predictable). In line with this approach, mean squares above 1.5 were rigorously excluded from the subscale. Items with mean square fits below 0.5 were accepted only for inclusion for content purposes. Besides the optimization on item level, we applied the Andersen likelihood ratio test with the median of age and sex as split criteria on all items with acceptable fits on item level [57]. The Andersen likelihood ratio test is a powerful test for differential responding, thus to find out whether younger and older individuals (or women and men) at the same exposure level would report items with a different probability. If the Andersen likelihood ratio test yielded significant results, we calculated the Wald test for each item of the subscale and checked if removing the item would prevent differential responding of the subscale for younger and older individuals or women and men. Finally, we aimed to identify individuals with above threshold exposure. Therefore, we tried to find the optimal discrimination of the subscale between moderate to severe levels of exposure, as indicated by the highest amount of information on the overall test function between logit scores 0 and 2.

#### Test-retest reliability

To determine test-retest reliability, 14 participants completed the KERF-40-I twice. Spearman correlation coefficients were calculated with a two-sided significance. Procedure and results are reported in the S1 File in more detail.

#### Convergent validity

To measure the convergent validity, we used Pearson correlation coefficients to analyze the associations between global scores and subscale scores of the KERF-40+ and the well-established CTQ. In line with Fosse et al. [8], we expected that convergent validity would manifest as Pearson correlation coefficients above .6. First, the CTQ sum score was correlated with the KERF-40+ sum score including all subscales, and an additional KERF-40+ sum score including only the six KERF-40+ subscales corresponding to the five CTQ subscales (i.e., Parental Emotional Abuse, Parental Physical Abuse, Emotional Neglect, Physical Neglect, Sexual Abuse by a Member of the Household, and Sexual Abuse by Others Not Living in the Same Household). The KERF-40+ sum score encompassing only the six subscales corresponding to the five CTQ subscales is referred to as the KERF-40+ Sum-6. Second, we determined the ability of the KERF-40+ subscales to predict the presence and absence of above threshold exposure levels of childhood maltreatment as assessed with the corresponding CTQ subscales using receiver operating characteristic (ROC), sensitivity, and specificity analysis. To this aim, cutoffs were proposed indicating an above threshold level of exposure for each KERF-40+ subscale. For the six subscales of the KERF-40+ corresponding to five CTQ subscales, the cutoffs were identified as showing the highest sensitivity–specificity in predicting the presence and absence of the respective CTQ subscales [49]. Based on the cutoffs of the original version of the KERF [23], and considering the test information function on overall exposure levels, we proposed cutoffs for four subscales that did not correspond to any CTQ subscale (i.e., Physical and Emotional Abuse by Peers, Witnessed Violence towards Parents, Witnessed Violence towards Siblings, and Physical and Emotional Abuse by Siblings).

#### Predictive validity

To evaluate the relative predictive validity, Pearson correlation coefficients were calculated between global scores of the KERF-40+ (i.e., sum score, multiplicity score, and duration score) and the CTQ (i.e., sum score and multiplicity score), respectively, and total scores of psychometric measures of general and trauma-related psychopathology (i.e., BSI, BDI-II, PCL-5, FDS). Fisher *z* scores were used for within-sample comparisons between the correlation coefficients with psychometric measures of psychopathology with KERF-40+ and CTQ.

## Results

### KERF-40+ item to subscale assignment and scaling

Below, results are presented separately for each of the ten KERF-40+ subscales based on the total sample of *N* = 287 participants, including 206 participants with mental disorders and 81 healthy volunteers. Following the Rasch scaling procedure, three subscales of the original proposal have been revised: Emotional Abuse By Siblings (SEA), Physical Abuse by Siblings (SPA) and Sexual Abuse (SEXA). We could not identify a Rasch-based subscale for Physical Abuse by Siblings (SPA). Similarly to the subscale Physical and Emotional Abuse by Peers (PEER), and in order to select items from a larger pool, we decided to collapse items for physical and emotional abuse by siblings to the subscale Physical and Emotional Abuse by Siblings (PEAS). Furthermore, we failed to identify one Rasch-based subscale for sexual abuse by parents, siblings as well as other adults and peers not living in the same household. Thus, we decided to split the items into two subscales targeting sexual abuse by a member of the household (i.e., parents and siblings), and sexual abuse by others not living in the same household (i.e., adults and peers). Therefore, the Rasch model-based evaluation resulted in three new subscales: Physical and Emotional Abuse by Siblings (PEAS), Sexual Abuse by a Member of the Household (SEXA-H), and Sexual Abuse by Others Not Living in the Same Household (SEXA-O). Please refer to Table 2 for item numbers and item fit statistics of all subscales.

**Table 2:** Item fit statistics for all KERF-40+ subscales.

	Item difficulty β (SE)	Outfit MSQ	Infit MSQ
Parental Emotional Abuse (PEA)			
1A. Swore at you, called you names, insulted you	-0.56 (0.15)	0.61	0.70
2A. Yelled or screamed at you	-1.30 (0.16)	0.71	0.90
3A. Locked you in a closet, attic, basement or garage	2.12 (0.18)	1.16	0.82
4A. Threatened to leave or abandon you	1.16 (0.16)	1.09	1.00
5A. A parent was very difficult to please	-1.42 (0.17)	0.60	0.82
Parental Physical Abuse (PPA)			
6A. Intentionally pushed, grabbed, shoved, slapped, pinched, punched or kicked you	-1.27 (0.16)	0.71	0.81
7A. Spanked you with their open hand on your buttocks, arms or legs	-0.89 (0.15)	0.82	0.92
8A. Spanked you with an object such as a strap, belt, brush, paddle, rod, etc.	0.67 (0.15)	0.92	0.94
9A. Hit you so hard that you were physically injured	1.48 (0.18)	0.67	0.80
Physical and Emotional Abuse by Siblings (PEAS)			
1B. Swore at you, called you names, insulted you	-1.24 (0.22)	0.99	1.09
2B. Yelled or screamed at you	-0.04 (0.23)	0.85	0.89
6B. Intentionally pushed, grabbed, shoved, slapped, pinched, punched or kicked you	-1.34 (0.23)	0.71	0.84
8B. Spanked you with an object such as a strap, belt, brush, paddle, rod, etc.	1.21 (0.28)	0.82	0.93
9B. Hit you so hard, that you were physically injured	1.41 (0.30)	0.56	0.78
Emotional Neglect (EN)			
33. You felt that neither your mother nor your father were emotionally available to you	-0.72 (0.16)	0.90	0.98
34. You felt that neither your mother nor your father did have the time or interest to talk to you	1.08 (0.16)	1.18	1.16
*39. One or more individuals in your family made you feel loved	0.02 (0.15)	0.79	0.81
*40. One or more individuals in your family helped you feel important or special.	-0.38 (0.15)	0.80	0.85
Physical Neglect (PN)			
*35. One or more individuals in your family were there to take you to the doctor if needed	-0.47 (0.18)	0.86	0.86
36. You didn’t have enough to eat	0.33 (0.19)	0.99	0.99
37. You had to wear dirty clothes	1.54 (0.25)	0.52	0.64
*38. One or more individuals in your family were there to take care of you and protect you	-1.39 (0.19)	0.94	0.94
Witnessed Violence towards Parents (WITP)			
22. Saw adults living in the household push, grab, slap or throw something at your mother	-1.58 (0.21)	0.61	0.77
23. Saw adults living in the household hit your mother so hard that it left marks for more than a few minutes	-0.07 (0.20)	0.64	0.69
24. Saw adults living in the household push, grab, slap or throw something at your father	-0.11 (0.20)	1.14	1.13
25. Saw adults living in the household hit your father so hard that it left marks for more than a few minutes	1.75 (0.29)	0.92	0.84
Witnessed Violence towards Siblings (WITS)			
16. Threatened to harm your sibling	-0.38 (0.19)	1.03	1.00
17. Intentionally pushed, grabbed, shoved, slapped, pinched, punched, or kicked your sibling	-1.53 (0.20)	0.68	0.83
18. Spanked your sibling with their open hand on the buttocks, arms or legs	-0.54 (0.19)	0.84	0.94
19. Spanked your sibling with an object such as a strap, belt, brush, paddle, rod, etc.	0.71 (0.20)	0.71	0.80
20. Hit your sibling hard that he or she was physically injured	1.74 (0.24)	0.91	0.96
Physical and Emotional Abuse by Peers (PEER)			
26A. Swore at you, called you names, insulted you	-1.09 (0.16)	0.80	0.89
27A. Said things behind your back, posted derogatory messages about you, or spread rumors about you	-0.84 (0.15)	0.69	0.81
28A. Intentionally excluded you from activities or groups	-0.22 (0.15)	0.94	0.94
29A. Intentionally pushed, grabbed, shoved, slapped, pinched, punched, or kicked you	0.06 (0.15)	1.07	1.05
30A. Hit you so hard that you were physically injured.	2.09 (0.21)	0.90	0.81
Sexual Abuse by a Member of the Household (SEXA-H)			
10A. Touched or fondled your body in a sexual way	-1.10 (0.31)	1.03	1.02
11A. Had you touch their body in a sexual way	-0.36 (0.32)	0.82	0.86
12A. Had any type of sexual intercourse (oral, anal or vaginal) with you	0.29 (0.36)	0.58	0.72
10B. Your sibling touched or fondled your body in a sexual way	-0.36 (0.32)	1.35	1.30
11B. Had you touch the body (of your sibling) in a sexual way	0.45 (0.37)	1.12	1.02
12B. Your sibling had any type of sexual intercourse (oral, anal or vaginal) with you	1.08 (0.46)	0.58	0.80
Sexual Abuse by Others Not Living in the Same Household (SEXA-O)			
13. An adult not living in the house touched or fondled your body in a sexual way	-1.84 (0.20)	0.89	0.94
14. An adult not living in the house had you touch their body in a sexual way	-0.30 (0.21)	0.66	0.77
15. An adult not living in the house had any type of sexual intercourse (oral, anal or vaginal) with you.	-0.30 (0.21)	0.61	0.71
31A. A peer (not in a romantic relationship) forced you to engage in sexual activity against your will	-0.48 (0.20)	1.14	1.11
32A. A peer (not in a romantic relationship) forced you to do things sexually that you did not want to do.	1.29 (0.32)	1.22	1.06
31B. Someone in a romantic relationship forced you to engage in sexual activity against your will	0.59 (0.26)	1.25	1.04
32B. Someone in a romantic relationship forced you to do things sexually that you did not want to do	1.05 (0.30)	0.87	0.90

MSQ = mean square fits. SE = standard error.

*Subscale 1*. *Parental Emotional Abuse (PEA)*. The first five items of the KERF-40-I were considered for inclusion of this subscale. All mean square fits were below 1.3, even though two items obtained a small outfit of 0.61 for item 1A and 0.60 for item 5A, indicating some degree of item redundancy. The Andersen tests with age and sex as a split criterion were not significant, indicating fit to the Rasch model, *Χ*^2^(4) = 8.17, *p* = .086 (median of age as the split criterion), and *Χ*^2^(4) = 2.94, *p* = .567 (sex as the split criterion). The optimal discrimination was observed for the target range of logit scores between 0 and 2 (moderate to severe levels), including 35.1% of the total information.

*Subscale 2*. *Parental Physical Abuse (PPA)*. Four items (i.e., item 6A, 7A, 8A and 9A) were available for inclusion in this subscale. None of the four items showed an outfit above 1.3. Item 9A obtained a smaller outfit of 0.67, indicating some degree of item redundancy. Item 9A were kept in the subscale to meet the minimum number of items per subscale. The Andersen test was significant when splitting for age, *Χ*^2^(3) = 8.92, *p* = .030. The Wald test indicated differential item response patterns for younger and older individuals on item 6A (*Z* = 2.12, *p* = .034) and item 8A (*Z* = -2.5, *p* = .012). There was no differential item responding when splitting for sex, *Χ*^2^(3) = 1.51, *p* = .680. The highest information was found in the target level of exposure with 38.69% of the total information.

*Subscale 3*. *Physical and Emotional Abuse by Siblings (PEAS)*. Because we were unable to allocate items for physical abuse by siblings, we decided to collapse all items of physical and emotional abuse by siblings. Thus, nine items were available for the potential inclusion in the subscale. First, item 4B was excluded due to a high outfit of 3.05. Second, item 3B had to be removed from the scale due to a high outfit of 1.63. Third, item 5B had a high outfit of 1.47 and was removed. Fourth, item 7B was removed due to a lower outfit of 0.46. Subsequently, we achieved a subscale with five items including item 1B, 2B, 6B, 8B, and 9B. Item 9B had a small outfit of 0.56, indicating some degree of item redundancy. Both Andersen tests were not significant, *Χ*^2^(4) = 3,15, *p* = .534 (median of age as the split criterion), and *Χ*^2^(4) = 1.00, *p* = .910 (sex as the split criterion), indicating similar fits to the Rasch model for younger and older individuals as well as for women and men. The test information curve showed that the test best discriminated high exposure levels with logit scores between 2 and 4, including 40.02% of the information. In the target range (i.e., logit score between 0 and 2), this scale had 30.76% of the total information.

*Subscale 4*. *Emotional Neglect (EN)*. Four items were included (i.e., item 33, 34, 39, 40). Item 39 and item 40 were recoded prior to the inclusion in the subscale. None of the items showed a misfit to the scale and both Andersen tests were not significant, *Χ*^2^(3) = 4.56, *p* = .207 (median of age as the split criterion) and *Χ*^2^(3) = 6.11, *p* = .106 (sex as the split criterion). The test information function had the highest information of the scale within the target range between 0 and 2, indicating optimal discrimination between moderate to higher level of exposure with 41.13% of the total information.

*Subscale 5*. *Physical Neglect (PN)*. Four items were considered for the subscale physical neglect (i.e., item 35, 36, 37 and 38). Items 35 and item 38 were reversed prior to inclusion. Item 37 had a small outfit of 0.50, indicating some degree of redundancy. Due to the minimum number of items per subscale, this item was kept. The Andersen tests showed no differences in the Rasch models, neither for younger and older individuals nor for women and men, *Χ*^2^(3) = 6.20, *p* = .102 (median of age as the split criterion), and *Χ*^2^(3) = 4.44, *p* = .218 (sex as the split criterion). The test information curve had the highest information within the target range between 0 and 2, indicating optimal discrimination between moderate to higher level of exposure with 35.94% of the total information.

*Subscale 6*. *Witnessed Violence Towards Parents (WITP)*. Five items were available for this subscale (i.e., item 22, 23, 24, 25, and 41). We removed item 41 due to a very low outfit of 0.10, indicating high level of redundancy. Following the removal of item 41, we observed low outfits for two items that were still in the acceptable range (outfit of 0.61 for item 22, and outfit of 0.64 for item 23). In order to achieve the minimum number of items, both items were kept in the subscale. We did not find any differences for younger and older individuals as well as women and men as indicated by the non-significant Andersen tests, *Χ*^2^(3) = 2.36, *p* = .501 (median of age as the split criterion), and *Χ*^2^(3) = 0.83, *p* = .843 (sex as the split criterion). The test information curve indicated the highest level of information for high levels of exposure (37.33%). In the target range of moderate to severe levels lay 32.09% of the information.

*Subscale 7*. *Witnessed Violence Towards Siblings (WITS)*. Initially, six items were available for the potential inclusion of the subscale (i.e., item 16, 17, 18, 19, 20, 21). Item 21 had an outfit of 6.09, indicating randomness for this subscale. After removing this item, none of the items had an outfit above 1.3. For item 17 an outfit of 0.68 was observed. The Andersen test showed different item functions for younger and older individuals, *Χ*^2^(4) = 16.52, *p* = .002. The Wald test indicated differential response patterns for younger and older individuals for item 16 (*Z* = 3.00, *p* = .003), item 17 (*Z* = 2.03, *p* = .042), item 18 (*Z* = -2.22, *p* = .027), and item 19 (*Z* = -2.12, *p* = .034). There was no significant difference between women and men, *Χ*^2^(4) = 1.68, *p* = .794. The removal of any item did not improve the subscale, therefore we decided to keep all items in the subscale. The test information curve revealed the optimal discrimination of the test in the target range (i.e., logit score between 0 and 2) with 41.78% of the total information.

*Subscale 8*. *Physical and Emotional Abuse by Peers (PEER)*. Five items were eligible for inclusion in the subscale (i.e., item 26A, 27A, 28A, 29A and 30A). With the exception of the small outfit of item 27A, all other fits scores were in the target range. The Andersen test indicated differences for younger and older individuals, *Χ*^2^(4) = 11.06, *p* = .026. Item 28A showed a differential response pattern for younger and older individuals (*Z* = 2.05, *p* = .012). When comparing women and men, the Andersen test also indicated differential item responses, *Χ*^2^(4) = 27.69, *p* < .001. The Wald test revealed differential response patterns for women and men for item 27A (*Z* = 2.37, *p* = .018), item 28A (*Z* = 2.55, *p* = .011), item 29A (*Z* = - 3.65, *p* < .001), and item 30A (*Z* = -2.31, *p* = .021). The test information curve showed the highest information in the target range of moderate to severe exposure levels with 38.4% of the total information.

*Subscale 9*. *Sexual Abuse by a Member of the Household (SEXA-H)*. There were six items for the inclusion of the scale (i.e., item 10A, 10B, 11A, 11B, 12A, and 12B). Item 10B indicated a high outfit of 1.35. In order to have the same number of items for sexual abuse by caregivers and siblings, we decided to keep item 10B. We were not able to calculate the Andersen test for younger and older individuals because the frequency of the items in the subgroups was too low. The Andersen test for women and men did not reveal a different Rasch model for women and men, *Χ*^2^(4) = 2.95, *p* = .567 (item 12A was excluded for the test due to the lack of positive responses for men). This subscale demonstrated optimal discrimination for severe exposure levels with 39.73% of the total information. In the target range, there was 13.15% of the total information.

*Subscale 10*. *Sexual Abuse by Others Not Living in the Same Household (SEXA-O)*. For this subscale, seven items were eligible (i.e., item 13, 14, 15, 31A, 32A, 31B, and 32B). Items 14 and 15 showed low outfits, pointing to some degree of redundancy. The Andersen test indicated no deviation from the Rasch model for younger and older individuals, *Χ*^2^(6) = 4.48, *p* = .613. There was no differential item response pattern for women and men, *Χ*^2^(5) = 5.48, *p* = .360 (item 32B was excluded from the test due to the lack of positive responses for men). This subscale demonstrated the optimal discrimination for severe exposure levels with 40.59% of the total information. In the target range, there was 25.37% of the total information.

### Test-retest reliability of the KERF-40+

Test-retest reliability had acceptable to excellent quality for the KERF-40+ global scores, including the KERF-40+ sum score (ρ = .88, *p* < .001), multiplicity score (ρ = .91, *p* < .001), and duration score (ρ = .74, *p* = .003). All ten subscales showed significant correlations between T1 and T2. However, nonsignificant shifts in the mean values were observed from T1 to T2, showing lower values for T2 as compared to T1. See S1 File for more details.

### Convergent validity between KERF-40+ and CTQ

Convergent validity between the KERF-40+ and CTQ can generally be characterized as excellent. As presented in Table 3, analyses revealed a strong correlation between the KERF-40+ sum score and the CTQ sum score (*r* = .83, *p* < .001). The strength of the correlation increased when the KERF-40+ sum score was adjusted to match the five subscales of the CTQ (i.e., KERF-40+ Sum-6; *r* = .87, *p* < .001). Additional analyses yielded strong correlations between corresponding KERF-40+ and CTQ subscale scores (*r*’s ≥ .56, *p* < .001; please see Table 4 and S2 Table).

**Table 3:** Convergent validity, as indicated by correlations between KERF-40+ global scores and CTQ sum score.

	KERF-40+ Sum	KERF-40+ Sum-6	KERF-40+ Multiplicity	KERF-40+ Duration	CTQ Sum
KERF-40+ Sum	1				
KERF-40+ Sum-6	.92***	1			
KERF-40+ Multiplicity	.95***	.89***	1		
KERF-40+ Duration	.77***	.76***	.77***	1	
CTQ Sum	.83***	.87***	.83***	.72***	1

Data are presented as Pearson correlation coefficients. CTQ = Childhood Trauma Questionnaire.

*** *p* < .001

**Table 4:** Receiver operating characteristic, sensitivity and specificity analysis of the KERF-40+.

KERF-40+ subscale	Number of items (item weight)	Corresponding CTQ subscale	Correlation with corresponding CTQ subscale	ROC area	Proposed cutoff	Sensitivity / specificity
Parental Emotional Abuse (PEA)	5 (2)	Emotional Abuse	.77	.89	3	.84 / .84 (*n* = 282)
Parental Physical Abuse (PPA)	4 (2.5)	Physical Abuse	.73	.94	3	.90 / .85 (*n* = 281)
Physical and Emotional Abuse by Siblings (PEAS)	5 (2)	-	-	-	3	
Emotional Neglect (EN)	4 (2.5)	Emotional Neglect	.76	.86	2	.84 / .78 (*n* = 282)
Physical Neglect (PN)	4 (2.5)	Physical Neglect	.78	.87	2	.62 / .95 (*n* = 282)
Witnessed Violence towards Parents (WITP)	4 (2.5)	-	-	-	2	
Witnessed Violence towards Siblings (WITS)	5 (2)	-	-	-	3	
Physical and Emotional Abuse by Peers (PEER)	5 (2)	-	-	-	2	
Sexual Abuse by a Member of the Household (SEXA-H)	6 (1.67)	Sexual Abuse	.66	.71	1	.43 / .99 (*n* = 282)
Sexual Abuse by Others Not Living in the Same Household (SEXA-O)	7 (1.43)	Sexual Abuse	.56	.83	1	.79 / .80 (*n* = 281)

The correlations between the corresponding KERF-40+ and CTQ subscales are presented as Pearson correlation coefficients. CTQ = Childhood Trauma Questionnaire. ROC = Receiver operating characteristic.

Results of the ROC, sensitivity, and specificity analysis of the KERF-40+, as well as proposed cutoffs are summarized in Table 4. Four of the ten KERF-40+ subscales did not correspond to any of the five CTQ subscales (i.e., Physical and Emotional Abuse by Siblings, Physical and Emotional Abuse by Peers, Witnessed Violence towards Parents, Witnessed Violence towards Siblings). Based on the cutoffs of the original version of the KERF [23], and considering the distribution of the test information function, we proposed a cutoff of 2 for the subscales WITP and PEER, and a cutoff of 3 for the subscales PEAS and WITS.

### Relative predictive validity of the KERF-40+ as compared to the CTQ

As indicated in Table 5, sum scores and multiplicity scores of both KERF-40+ and CTQ were significantly correlated with total scores of the BSI (BSI GSI), BDI-II, PCL-5, and FDS. Follow-up comparisons of within-group correlations using Fisher *z* scores revealed that the size of the correlation coefficients generally did not differ significantly between KERF-40+ and CTQ. In one-sided tests (*z* ≥ 1.65), the PCL-5 sum score had a significantly stronger association with the CTQ sum score and CTQ multiplicity score, as compared to the KERF-40+ sum score (*z* = -1.88, *p* = .030) and KERF-40+ multiplicity score (*z* = -1.69, *p* = .046), respectively, and the FDS DES score showed a significantly stronger association with the CTQ sum score, as compared to the KERF duration score (*z* = -1.76, *p* = .039). In two-sided tests (*z* ≥ 1.96), no significant differences were detected between KERF-40+ and CTQ with regard to the correlation coefficients with psychometric measures of psychopathology (i.e., BSI, BDI-II, PCL-5, FDS).

**Table 5:** Relative predictive validity, as indicated by correlations between KERF-40+ scores, CTQ scores and psychometric measures of psychopathology.

	Sum Scores			Multiplicity Scores	
	KERF-40+ Sum	KERF-40+ Duration	CTQ Sum	KERF-40+ Multi	CTQ Multi
General psychopathology (BSI GSI; *n* = 224)	.33***	.36***	.36***	.29***	.35***
Depressiveness (BDI-II sum score; *n* = 195)	.45***	.42***	.43***	.40***	.40***
PTSD symptom severity (PCL-5 sum score; *n* = 223)	.44***	.44***	.50***	.42***	.48***
Dissociation (FDS DES score; *n* = 218)	.27***	.24***	.33***	.27***	.31***

Data are presented as Pearson correlation coefficients. BDI-II = Beck Depression Inventory, revised version, BSI GSI = Brief Symptom Inventory Global Severity Index, FDS = Fragebogen für dissoziative Symptome (German adaptation of the Dissociative Experience Scale, DES), PCL-5 = PTSD Checklist for DSM-5, PTSD = posttraumatic stress disorder.

*** *p* < .001

### Descriptive statistics of the KERF-40+

**Fig 1:**
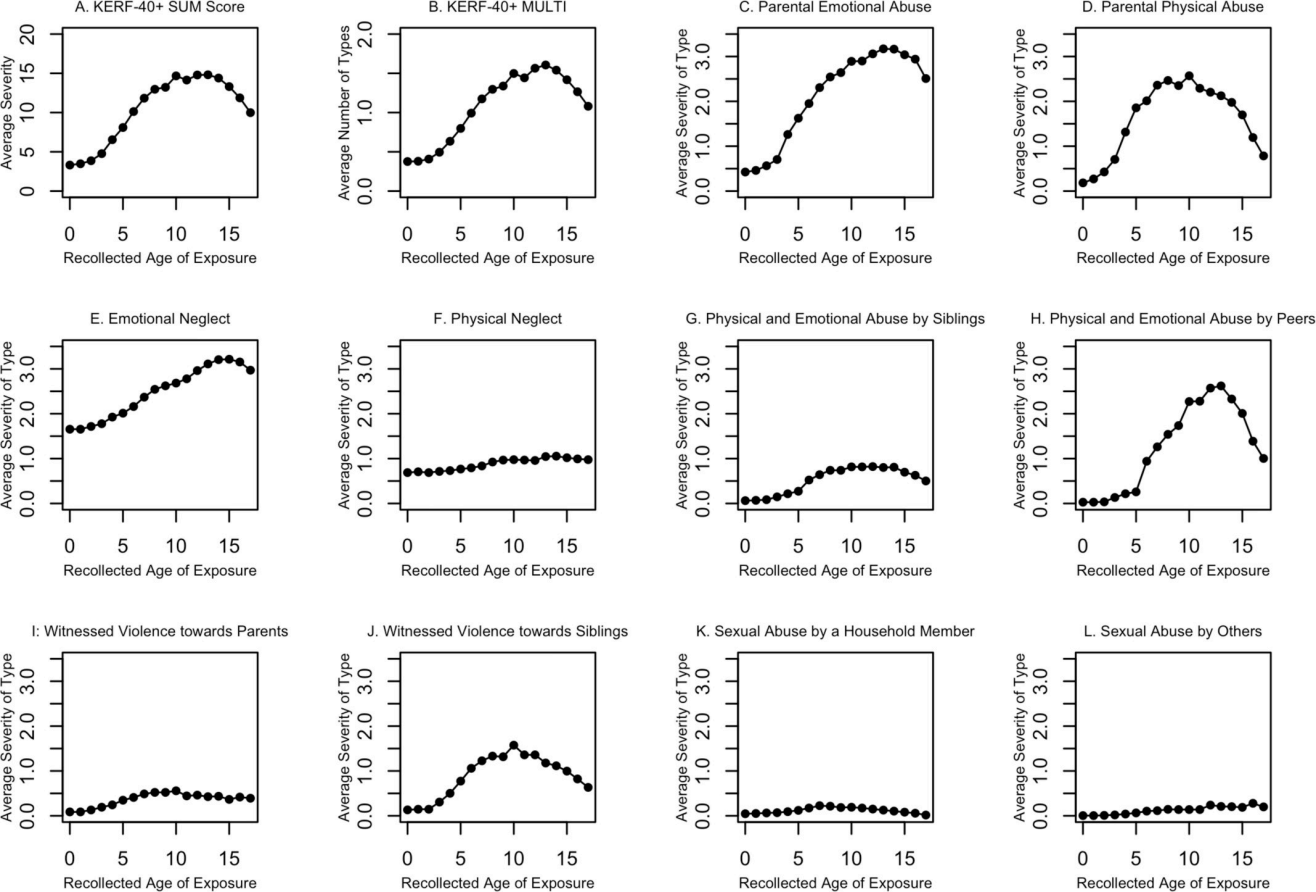
KERF-40+ global and subscale scores as a function of recollected age of exposure. Panel A and B show the time course of KERF-40+ global scores, including sum and multiplicity score, while panel C to L demonstrate the time course of the ten KERF-40+ subscales.

Most of the 287 participants were exposed to at least one type of childhood maltreatment (i.e., reported above the cutoff level) captured with the KERF-40+ (81.9%, *n* = 235). The most prevalent type of childhood maltreatment was Emotional Neglect (50.9%, *n* = 146), followed by Parental Emotional Abuse (47.4%, *n* = 136), Physical and Emotional Abuse by Peers (44.9%, *n* = 129), Sexual Abuse by Others Not Living in the Same Household (36.2%, *n* = 104), Parental Physical Abuse (32.4%, *n* = 93), Witnessed Violence towards Siblings (23.7%, *n* = 68), Physical Neglect (20.6%, *n* = 59), Witnessed Violence Towards Parents (17.4%, *n* = 50), Sexual Abuse by a Member of the Household (12.2%, *n* = 35), and Physical and Emotional Abuse by Siblings (9.4%, *n* = 27). With regard to the timing of childhood maltreatment, the peak for KERF-40+ sum score and KERF-40+ multiplicity score was observed at age 13 (see Fig 1 for the recollected age of exposure of the KERF-40+ global and subscale scores).

## Discussion

In this study, a brief German interview version of the MACE, the KERF-40-I, was validated in a sample of 287 participants with and without mental disorders, leading to the development of the KERF-40+. Our item to subscale and scaling analysis yielded ten different subscales, capturing ten different types of childhood maltreatment with a total of 49 items. In addition to commonly assessed types of childhood maltreatment (i.e., parental emotional abuse, parental physical abuse, sexual abuse by members of one’s own household or by others not living in the same household, emotional neglect, physical neglect), the KERF-40+ allows for measuring further important, but understudied types of childhood maltreatment, such as emotional and physical abuse by siblings or by peers as well as witnessing violence towards parents or siblings. In addition, the KERF-40+ allows to assess the timing and duration of different types of childhood maltreatment.

Subsequent to our item to subscale and scaling analysis, Andersen likelihood ratio tests showed that for the ten KERF-40+ subscales, person homogeneity was generally not violated across age and sex. However, significant differences were revealed between younger and older participants regarding parental physical abuse, witnessing violence towards siblings, as well as physical and emotional abuse by peers. Moreover, significant differences were detected between male and female participants regarding physical and emotional abuse by peers. First, follow-up tests indicated that younger and older participants responded differently when being asked about their parents “intentionally pushing, grabbing, shoving, slapping, pinching, punching, or kicking them” (item 6A) and “spanking them with an object” (item 8A) up to the age of 18 years. Second, follow-up tests showed differential response patterns for younger and older participants when being asked about whether they witnessed their parents being violent towards their sibling(s) in terms of “threatening to harm their sibling(s)” (item 16), “intentionally pushing, grabbing, shoving, slapping, pinching, punching, or kicking their sibling(s)” (item 17), “spanking their sibling(s) with their open hand on the buttocks, arms, or legs” (item 18), and “spanking their sibling(s) with an object” (item 19) up to the age of 18 years. Differential age-dependent response patterns concerning experiences of parental physical abuse towards oneself and one’s sibling(s) might reflect changes in child-rearing practices [8]. Since January 1, 2001, the right of children to a non-violent upbringing has been integrated into the German civil code of law [§ 1631 Bürgerliches Gesetzbuch, “Gesetz zur Ächtung der Gewalt in der Erziehung”; 58]. Nowadays, a non-violent upbringing is perceived as an important social norm in Germany [58], and serious parental physical abuse has declined sharply in the last 20 years [59]. Although we cannot directly infer reasons for age-dependent differential response patterns based on our data, we may assume that changes in child-rearing practices may have contributed to the differences found between younger and older participants in our study. Third, follow-up tests showed that both younger and older participants, as well as male and female participants, responded differently regarding experiences of their peers “intentionally excluding them from activities or groups” (item 28A). Moreover, follow-up tests indicated that male and female participants exhibited differential response patterns regarding further experiences of physical and emotional peer abuse. Differential item response patterns across younger and older, as well as male and female participants may suggest that age- and sex-related differences should be considered when assessing physical and emotional peer victimization.

While needed to be interpreted with caution due to the limited sample size, stability of test-retest analyses was shown to be acceptable to excellent for both global scores and nine out of ten subscale scores, with the exception of the subscale targeting emotional neglect. Our results are mostly in line with studies on the US [9] and Norwegian [8] versions of the MACE, indicating excellent test-retest reliability for global scores over two weeks in 87 psychiatric outpatients [8], and over three months on average in 75 healthy volunteers [9]. Our results are also mostly consistent with earlier findings on the test-retest reliability of ten subscales of the US version of the MACE [9], indicating acceptable to excellent reliability for all KERF-40+ subscales, with the exception of emotional and physical neglect. We observed descriptive differences between measures at T1 and T2. Differences in the reported severity of childhood maltreatment have been discussed as a consequence of an increase in symptom load, e.g. distress or depressiveness, being associated with an increase in the reported maltreatment severity [60]. However, due to the time frame and sample size, the results of our test-retest analyses may not be discussed in further depth.

Regarding convergent validity between the KERF-40+ and CTQ, our correlational results are consistent with those determined by the US [9], Norwegian [8], and original German [23] versions of the MACE. In line with previous studies, our correlational analyses yielded a strong relationship between the KERF-40+ sum score and CTQ sum score (our study: *r* = .83; Teicher & Parigger, 2015: *r* = .74; Fosse et al., 2020: *r* = .81; Isele et al., 2014: *r* = .75). By calculating the KERF-40+ sum score considering only the six KERF-40+ subscales which corresponded to the five CTQ subscales, our correlational analyses yielded an even stronger relationship between both KERF-40+ and CTQ sum scores (our study: *r* = .87; Fosse et al., 2020: *r* = .84; Isele et al., 2014: *r* = .83). While both KERF-40+ sum scores (i.e., based on 10 or 6 subscales, respectively) and multiplicity score correlated strongly with the CTQ sum score (*r’s* ≥ .83), the KERF-40+ duration score correlated strongly, albeit numerically somewhat lower, with the CTQ sum score (*r* = .72). In accordance with Fosse et al. [8], this finding may indicate that assessing temporal aspects (i.e., timing and duration) of childhood maltreatment experiences may capture additional valuable information, complementing information on degree or severity of exposure.

With regard to relative predictive validity, both KERF-40+ global scores (i.e., sum, multiplicity, and duration score) and CTQ global scores (i.e., sum, and multiplicity score) correlated significantly with general and trauma-related measures of psychopathology. Correlational analyses yielded moderate to strong associations between KERF-40+ and CTQ global scores, and general psychopathology (i.e., BSI GSI), consistent with those found in earlier studies [8, 23]. Moreover, correlational analyses revealed moderate to strong associations between KERF-40+ and CTQ global scores, and measures of depression severity, PTSD symptom severity, and dissociation. Contrary to earlier studies with the US [9] and Norwegian [8] version of the MACE, however, the KERF-40+ global scores did not account for substantially more variance in mental health symptoms than the established CTQ. Discrepancies between current and earlier findings may be explained by clinical characteristics of our sample. While earlier studies focused on healthy participants [9] or a composite participant group of employees and psychiatric outpatients with moderately severe mental health problems [8], our current sample also included psychiatric inpatients with severe mental health conditions. Thus, in our study, the capability of the KERF-40+ in predicting a broad range of psychopathological symptoms was comparable to the CTQ, which is widely perceived as the gold standard in the assessment of childhood maltreatment.

Our study provides a psychometrically sound, shortened and revised version of the original German MACE [23], the KERF-40+, which allows to assess type, timing, and duration of different childhood maltreatment experiences. Our study is characterized by a number of strengths, including studying a sample of 287 participants with and without various mental disorders, applying an advanced statistical method of item response theory, and using different self-report measures to assess a broad range of psychopathological symptoms. Some limitations should, however, be acknowledged. First, the test-retest analysis was performed by different trained interviewers in only a small subsample with mostly a very short test-retest interval of one day. Future studies should investigate larger samples with a longer and standardized test-retest interval, e.g., seven days, when aiming for a robust assessment of test-retest reliability, including the test-retest reliability of the timing scores. Further, as current symptom load might interfere with the reported severity of childhood maltreatment [60], this aspect should be accounted for if a larger time span lies between the two assessment time points. Second, the relative predictive validity was calculated only with self-report measures of psychopathology while neglecting clinician-administered rating scales of general or trauma-related symptomatology. Third, with regard to convergent validity, it would have been desirable to conduct both the KERF-40+ and the original KERF in the same sample to analyze the association between both measures, especially considering the four subscales that do not correspond to the CTQ (i.e., Physical and Emotional Abuse by Siblings, Witnessed Violence towards Parents, Witnessed Violence towards Siblings, Physical and Emotional Abuse by Peers). This limitation is particularly important given that the Physical and Emotional Abuse by Siblings subscale was not part of the original MACE scale [9] but was added in the KERF-40-I. Although previous research has emphasized the need to study sibling violence [13, 15, 17], our current findings indicate that this was the least endorsed type of childhood maltreatment. Therefore, we believe that further research is needed to determine the extent and impact of sibling victimization. Fourth, the cutoff scores for four subscales (i.e., Physical and Emotional Abuse by Peers, Witnessed Violence towards Parents, Witnessed Violence towards Siblings, Physical and Emotional Abuse by Siblings) were determined based on scores of the KERF [23], as well as the test information function of the respective subscale. In future studies, appropriate measures considering these types should be applied and converged with these subscales.

Taken together, the KERF-40+ represents a scaled and valid measure of type, timing, and duration of different childhood maltreatment experiences. While the CTQ allows to briefly screen for five core types of childhood maltreatment (i.e., emotional, physical, and sexual abuse as well as emotional and physical neglect), the KERF-40+ appears suitable to measure additional types of childhood maltreatment, their timing and duration. With recent research increasingly focusing on stress-sensitive developmental periods [e.g., 18, 20], the KERF-40+ constitutes a recommendable choice for retrospectively assessing severity, type and timing of childhood maltreatment experiences in samples of adult individuals with and without mental health disorders. Recent research highlights that taking timing and duration of childhood maltreatment experiences into account may considerably advance our current knowledge about the association between childhood maltreatment experiences and health outcomes later in life [20], emphasizing once again the need for comprehensive instruments such as the KERF-40+. The KERF-40-I on which the current evaluation proposal, the KERF-40+, is based, will be freely available under https://doi.org/10.23668/psycharchives.8151 from December 2022 onwards.

## Supporting information

S1 Table Comparison of the KERF-40-I and the KERF-40+. (DOCX)

S2 Table Correlations between corresponding KERF-40+ and CTQ subscales. (DOCX)

S1 Fig Figures from Rasch analyses for each KERF40+ subscale. (PDF)

S1 File Test-retest reliability of the KERF-40+. (DOCX)
