# Supplementary material for: Measuring types and timing of childhood maltreatment: The psychometric properties of the KERF-40+
Source: PLoS One. 2022 Sep 8;17(9):e0273931. doi: 10.1371/journal.pone.0273931 (PMC9455860; doi:10.1371/journal.pone.0273931)
Supplement: S1 Table — (DOCX) [file pone.0273931.s001.docx]

**S1 Table. Comparison of the KERF-40-I and the KERF-40+**

| Subscale | KERF-40-I | KERF-40+ | Comments |
| --- | --- | --- | --- |
| Original: Parental Emotional Abuse (PEA) | items 1A, 2A, 3A, 4A, 5A | items 1A, 2A, 3A, 4A, 5A | identical |
| Original: Parental Physical Abuse (PPA) | items 6A, 7A, 8A, 9A | items 6A, 7A, 8A, 9A | identical |
| Original: Emotional Abuse by Siblings (SEA) | items 1B, 2B, 3B, 4B, 5B | - | failed to scale |
| Original: Physical Abuse by Siblings (SPA) | items 6B, 7B, 8B, 9B | - | failed to scale |
| New: Emotional and Physical Abuse by Siblings (PEAS) | - | items 1B, 2B, 6B, 8B, 9B | items of Emotional Abuse by Siblings and Physical Abuse by Siblings were collapsed into one subscale |
| Original: Emotional Neglect (EN) | items 33, 34, 39r, 40r | items 33, 34, 39r, 40r | identical |
| Original: Physical Neglect (PN) | items 35r, 36, 37, 38r | items 35r, 36, 37, 38r | identical |
| Original: Witnessed Violence towards Parents (WITP) | items 22, 23, 24, 25 | items 22, 23, 24, 25 | identical |
| Original: Witnessed Violence towards Siblings (WITS) | items 16, 17, 18, 19, 20, 21 | items 16, 17, 18, 19, 20 | item 21 not included in the KERF-40+ |
| Original: Physical and Emotional Abuse by Peers (PEER) | items 26A, 27A, 28A, 29A, 30A | items 26A, 27A, 28A, 29A, 30A | identical |
| Original: Sexual Abuse | items 10A, 11A, 12A, 10B, 11B, 12B, 13, 14, 15, 31A, 32A | - | failed to scale |
| New: Sexual Abuse by a Member of the Household (SEXA-H) | - | items 10A, 11A, 12A, 10B, 11B, 12B | in the KERF-40+, we successfully scaled sexual abuse by members of the household (i.e., parents or siblings) |
| New: Sexual Abuse by Others Not Living in the Same Household (SEXA-O) | - | items 13, 14, 15, 31A, 32A, 31B, 32B | in the KERF-40+, we successfully scaled sexual abuse by others (i.e., adults not living in the same household or peers) |

*Note*. This table includes the similarities and differences between the KERF-40-I and the KERF-40+. For details, please refer to the main manuscript.
