# Supplementary material for: Measuring types and timing of childhood maltreatment: The psychometric properties of the KERF-40+
Source: PLoS One. 2022 Sep 8;17(9):e0273931. doi: 10.1371/journal.pone.0273931 (PMC9455860; doi:10.1371/journal.pone.0273931)
Supplement: S2 Table — (DOCX) [file pone.0273931.s002.docx]

**S4 Table. Correlations between KERF-40+ and CTQ subscales.**

|  |  | 1 | 2 | 3 | 4 | 5 | 6 | 7 | 8 | 9 | 10 | 11 |
| --- | --- | --- | --- | --- | --- | --- | --- | --- | --- | --- | --- | --- |
| 1 | KERF-40+ Parental Emotional Abuse | 1 |  |  |  |  |  |  |  |  |  |  |
| 2 | KERF-40+ Parental Physical Abuse | .60*** | 1 |  |  |  |  |  |  |  |  |  |
| 3 | KERF-40+ Sexual Abuse by a Member of the Household | .19** | .20** | 1 |  |  |  |  |  |  |  |  |
| 4 | KERF-40+ Sexual Abuse by Others Not Living in the Same Household | .21*** | .26*** | .27*** | 1 |  |  |  |  |  |  |  |
| 5 | KERF-40+ Emotional Neglect | .53*** | .36*** | .20** | .23*** | 1 |  |  |  |  |  |  |
| 6 | KERF-40+ Physical Neglect | .43*** | .36*** | .32*** | .30*** | .56*** | 1 |  |  |  |  |  |
| 7 | CTQ Emotional Abuse | **.77***** | .58*** | .33*** | .39*** | .61*** | .55*** | 1 |  |  |  |  |
| 8 | CTQ Physical Abuse | .55*** | **.73***** | .33*** | .40*** | .35*** | .51*** | .63*** | 1 |  |  |  |
| 9 | CTQ Sexual Abuse | .23*** | .26*** | **.66***** | **.56***** | .22*** | .39*** | .40*** | .46*** | 1 |  |  |
| 10 | CTQ Emotional Neglect | .62*** | .48*** | .31*** | .28*** | **.76***** | .59*** | .74*** | .52*** | .34*** | 1 |  |
| 11 | CTQ Physical Neglect | .48*** | .44*** | .37*** | .32*** | .55*** | **.78***** | .60*** | .59*** | .44*** | .68*** | 1 |

*Note*. Correlations between corresponding KERF-40+ and CTQ subscales are in bold. CTQ = Childhood Trauma Questionnaire.

** *p* < .01, *** *p* < .001
