# Supplementary material for: Measuring types and timing of childhood maltreatment: The psychometric properties of the KERF-40+
Source: PLoS One. 2022 Sep 8;17(9):e0273931. doi: 10.1371/journal.pone.0273931 (PMC9455860; doi:10.1371/journal.pone.0273931)

S2 Figures from Rasch analyses for each KERF-40+ subscale

Item Characteristic Curves, Item Information and Test Information Function for each Subscale

Figure A. Parental Emotional Abuse (PEA)

Figure B. Parental Physical Abuse (PPA)

Figure C. Physical and Emotional Abuse by Siblings (PEAS)

Figure D. Emotional Neglect (EN)

Figure E. Physical Neglect (PN)

Figure F. Witnessed Violence towards Parents (WITP)

Figure G. Witnessed Violence towards Siblings (WITS)

Figure H. Physical and Emotional Abuse by Peers (PEER)

Figure I. Sexual Abuse by a Member of the Household (SEXA-H)

Figure J. Sexual Abuse by Others Not Living in the Same Household (SEXA-O)

Figure A. Parental Emotional Abuse (PEA)

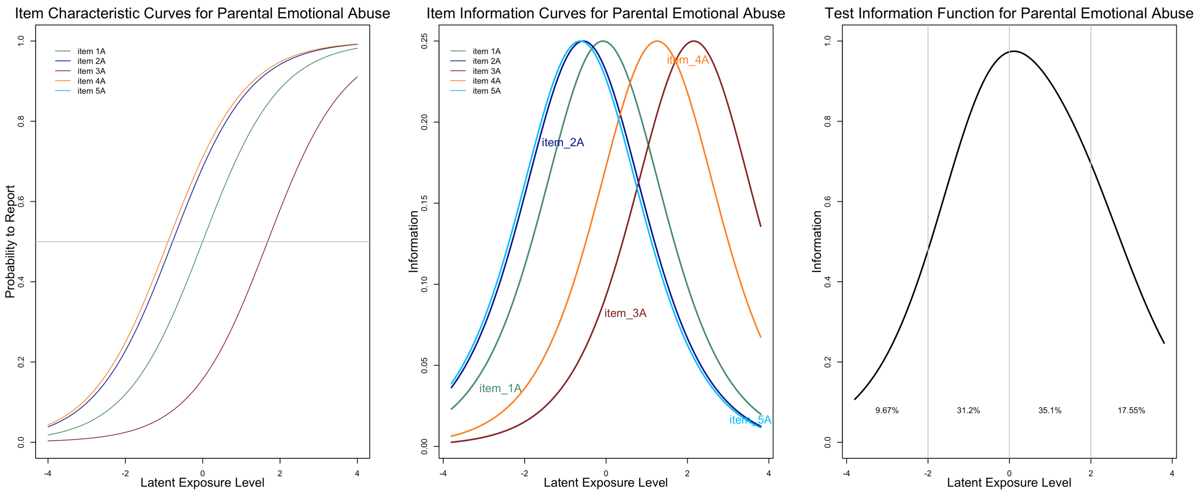

Figure B. Parental Physical Abuse (PPA)

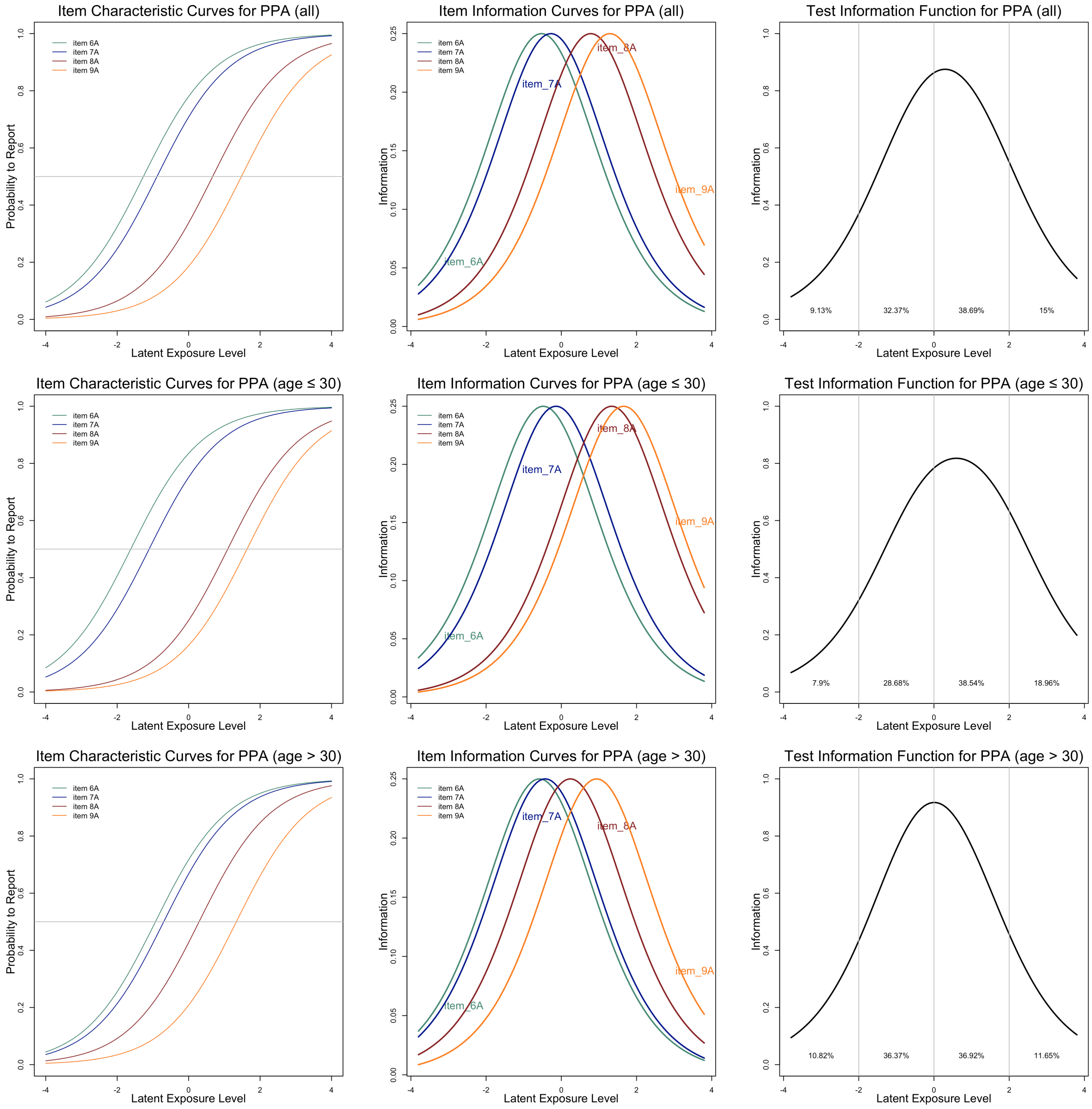

Figure C. Physical and Emotional Abuse by Siblings (PEAS)

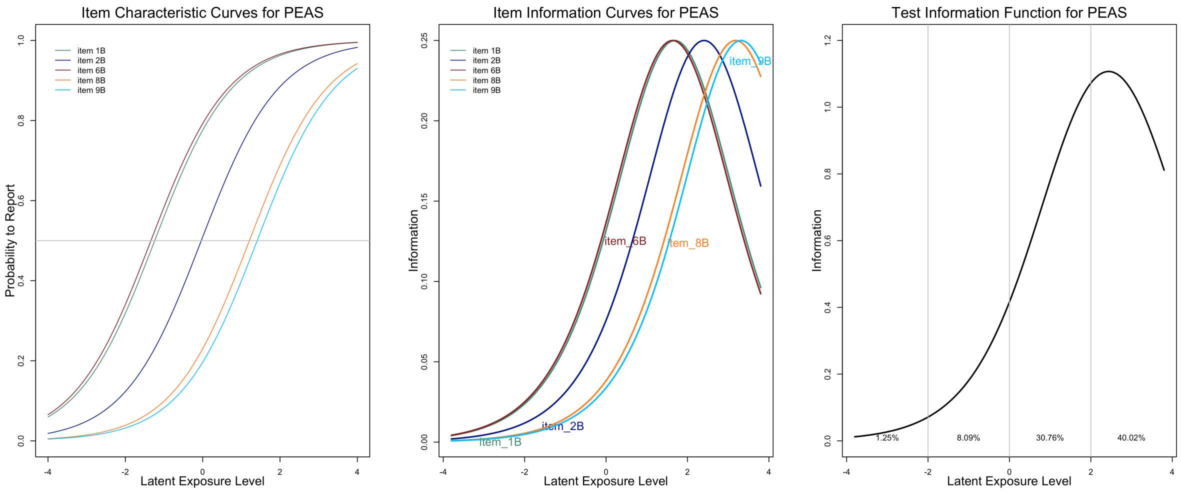

Figure D. Emotional Neglect (EN)

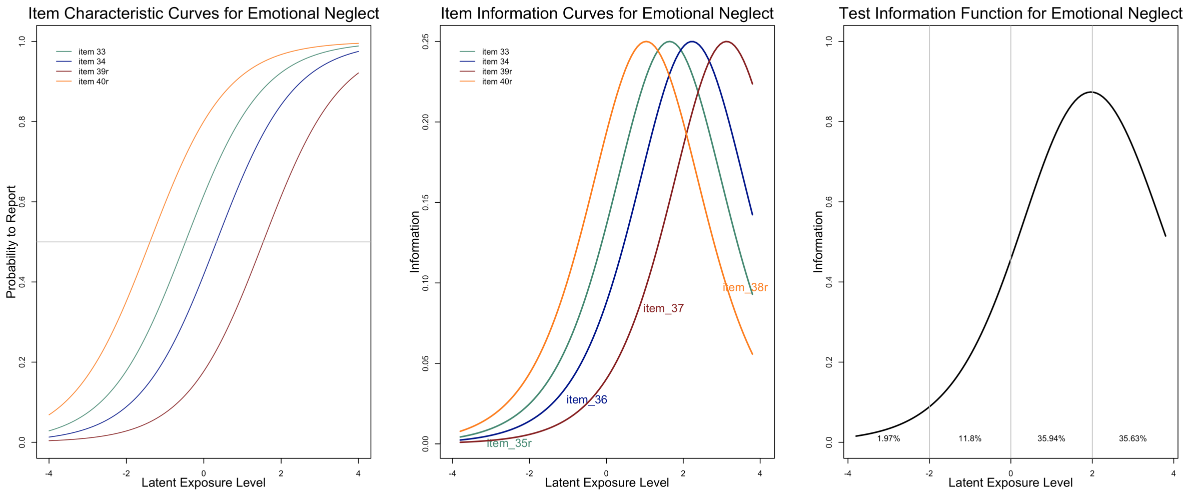

Figure E. Physical Neglect (PN)

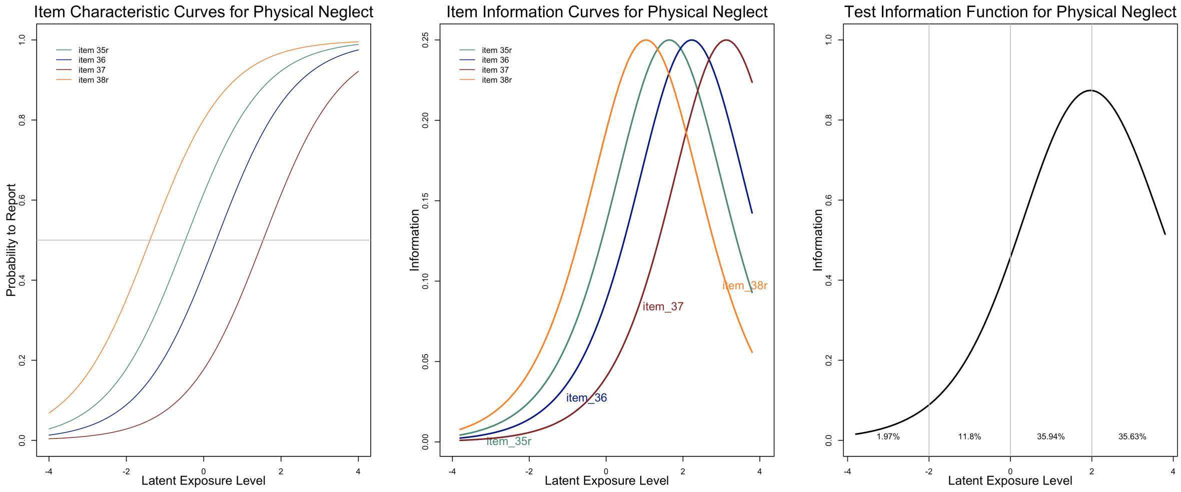

Figure F. Witnessed Violence towards Parents (WITP)

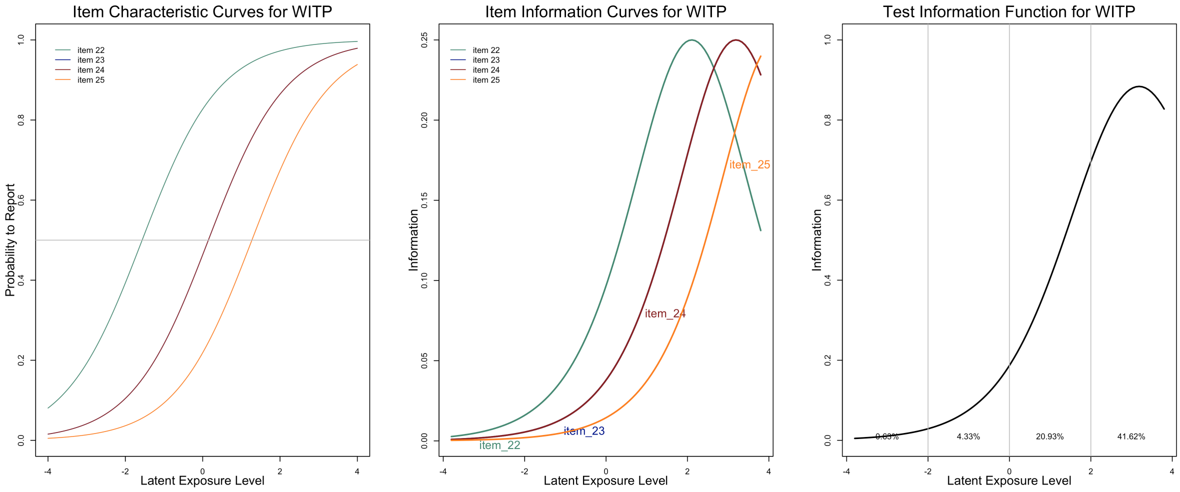

Figure G. Witnessed Violence towards Siblings (WITS)

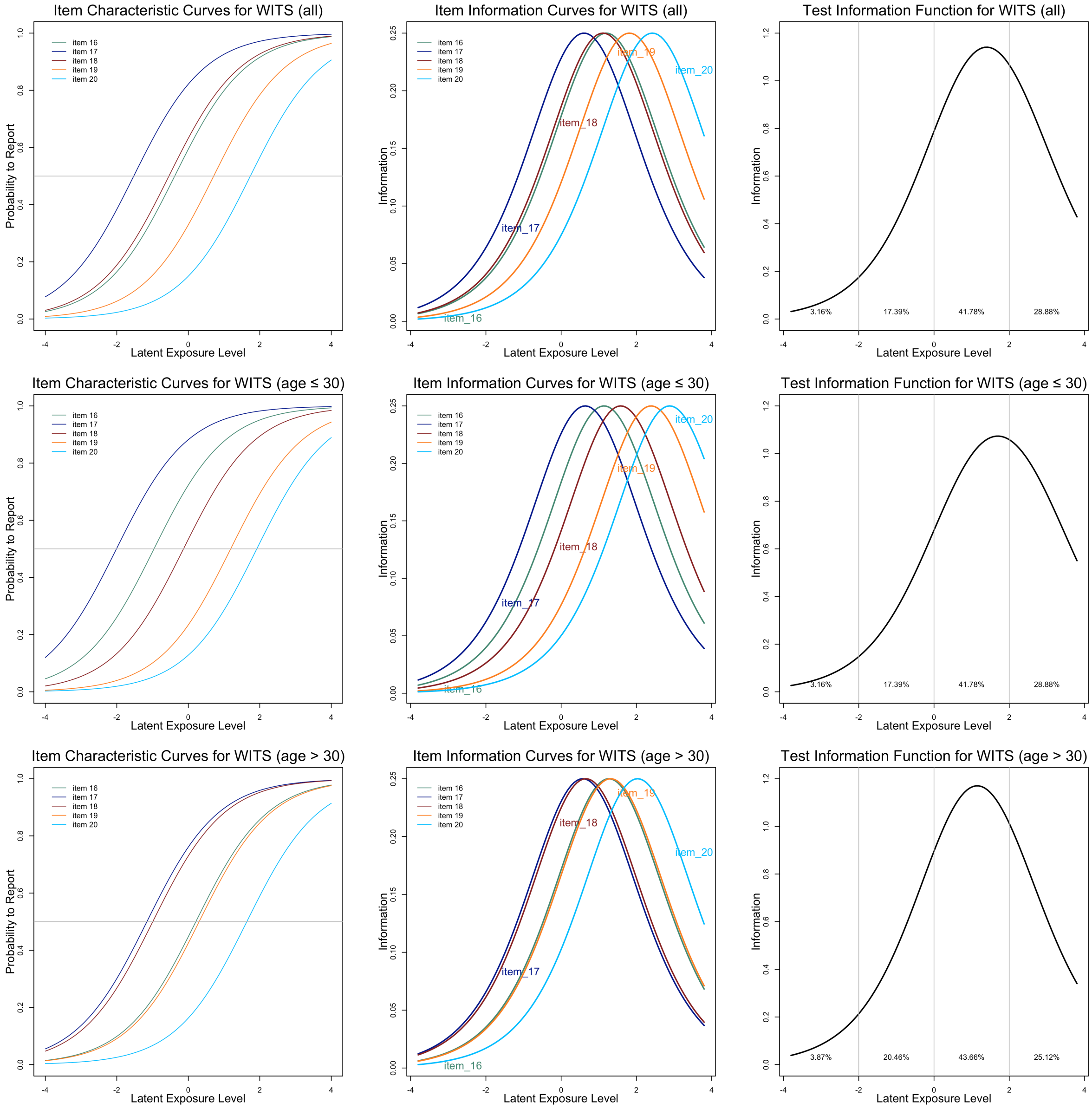

Figure H. Physical and Emotional Abuse by Peers (PEER)

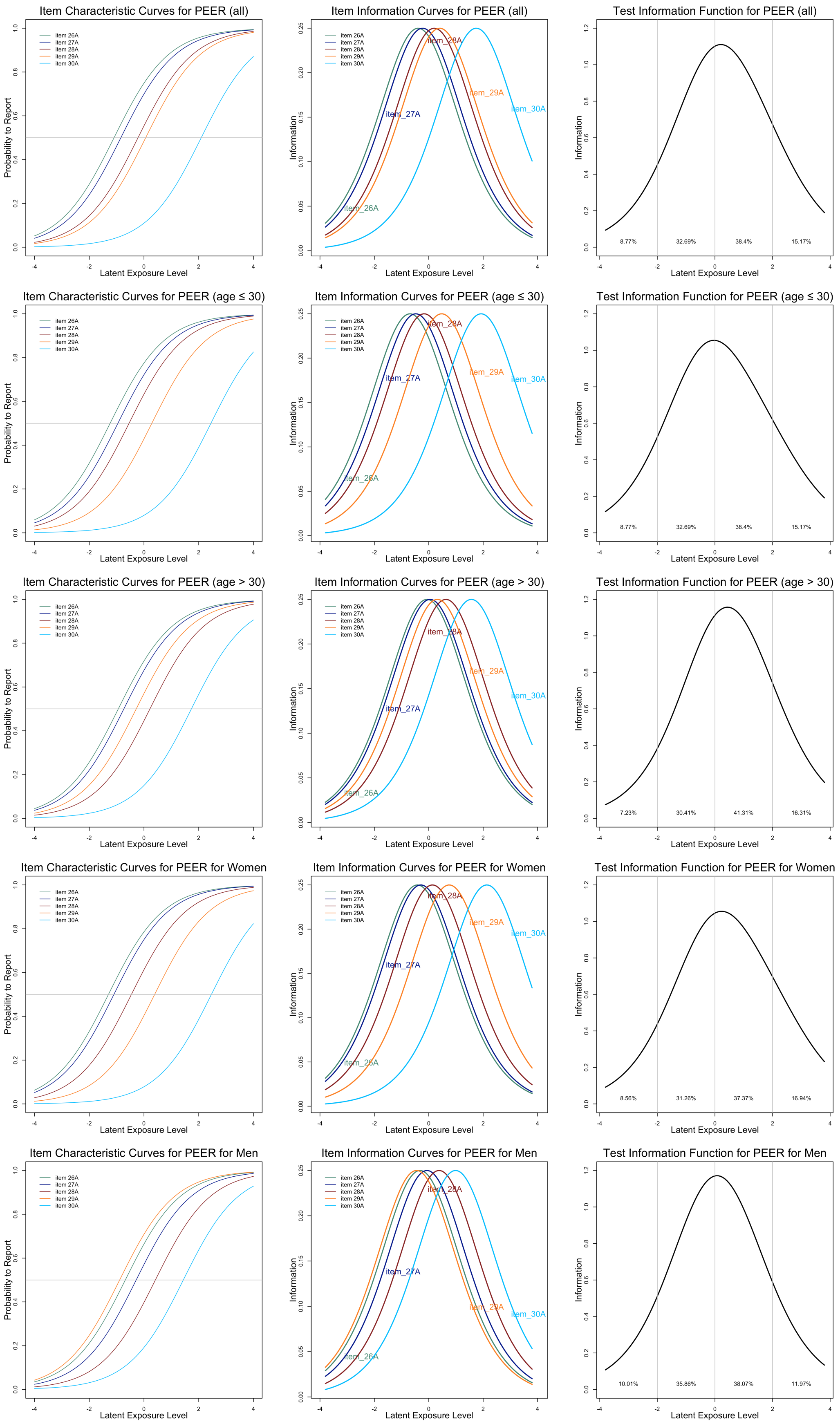

Figure I. Sexual Abuse by a Member of the Household (SEXA-H)

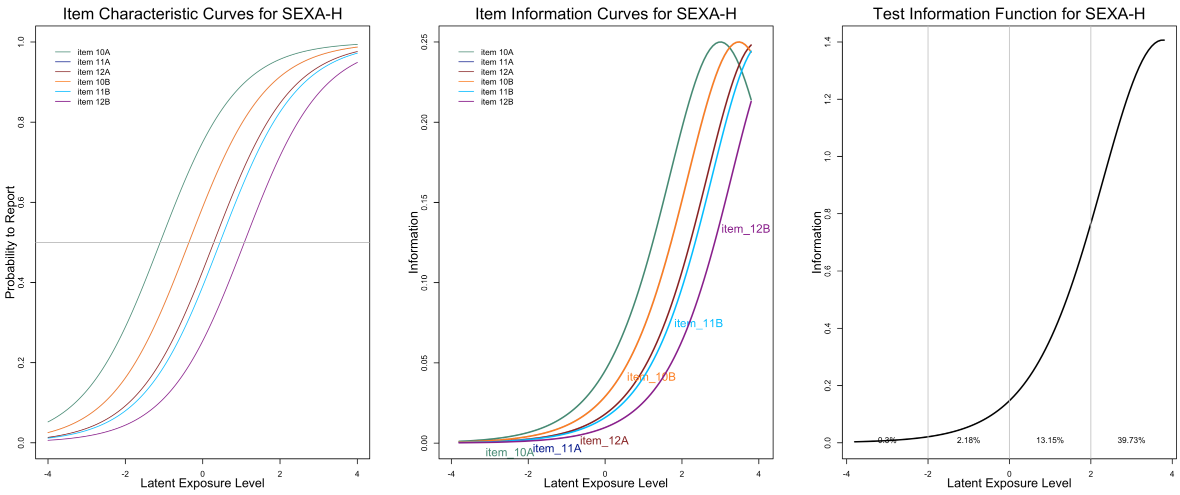

Figure J. Sexual Abuse by Others Not Living in the Same Household (SEXA-O)

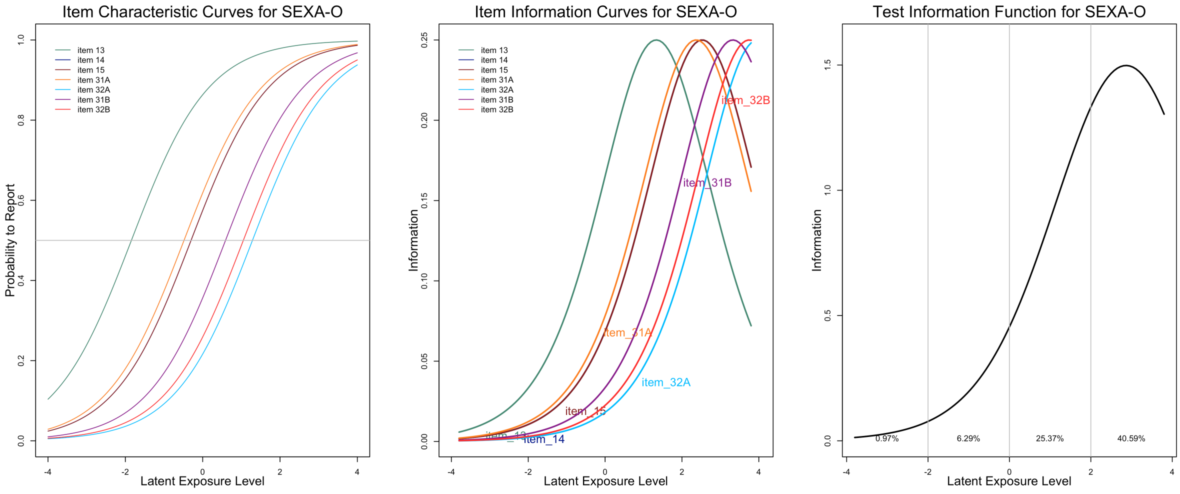

Supplement: S1 Fig — (PDF) [file pone.0273931.s003.pdf]
