# Supplementary material for: Measuring types and timing of childhood maltreatment: The psychometric properties of the KERF-40+
Source: PLoS One. 2022 Sep 8;17(9):e0273931. doi: 10.1371/journal.pone.0273931 (PMC9455860; doi:10.1371/journal.pone.0273931)
Supplement: S1 File — (DOCX) [file pone.0273931.s004.docx]

**Test-retest reliability of the KERF-40+**

To determine test-retest reliability, a small subsample of 14 participants (1 male; mean age 34.08 ± 10.71, range 21-53 years) completed the KERF-40-I twice with an inter-test interval of 1 day (*n* = 9) or 138 to 301 days (*n* = 5), respectively. Different interviewers from a pool of seven trained staff members performed test and re-test. Due to the recruitment procedure, participants participating in studies at different sites (i.e., Mannheim and Heidelberg) had longer test-retest intervals. Systematic reporting of severity at the first compared to the second examination time point was assessed using paired t-tests. Spearman’s correlation coefficients were calculated with a two-sided significance. Coefficients between .7 and .8, between .8 and .9, and between .9 and 1.0 were defined as acceptable, good, and excellent reliability, respectively. Test-retest analyses included KERF40+ global scores (i.e., sum score, multiplicity score, and duration score), as well as ten subscale scores. Additionally, Bland and Altman analyses of test-retests reliability were performed for the KERF-40+ global scores.

As shown in Supplementary Table S3, test-retest reliability had acceptable to excellent quality for the KERF-40+ global scores, including the KERF-40+ sum score (ρ = .88, *p* < .001), multiplicity score (ρ = .91, *p* < .001), and duration score (ρ = .74, *p* = .003). All ten subscales showed significant correlations between T1 and T2; correlation coefficients ranged between .55 (Emotional Neglect) and 1.00 (Sexual Abuse by a Member of the Household). Performing partial correlations including a dichotomous variable that indicated short (i.e., 1 day) and long (i.e., between 138 and 301 days) test-retest intervals, these results remained stable. However, nonsignificant shifts in the mean values were observed from T1 to T2, showing lower values for T2 as compared to T1 (see Supplementary Table S3). Bland and Altman analyses of reliability of test-retest scores for the KERF-40+ global scores (i.e., sum, duration, and multiplicity scores) are displayed in the Supplementary Figures S3A-3C.

**S3 Table. Test-retest reliability, as indicated by Spearman’s correlation coefficients and paired t-tests of KERF-40+ global and subscale scores.**

| KERF-40+ score | Mean (*SD*) T1 | Mean (*SD*) T2 | Spearman‘s rho | *p*-value | T1 vs. T2 | *p*-value |
| --- | --- | --- | --- | --- | --- | --- |
| Sum Score | 31.22 (16.9) | 28.43 (16.6) | .88 | <.001 | *t*(13)= 1.47 | .166 |
| Multiplicity Score | 3.86 (2.2) | 3.57 (2.2) | .91 | <.001 | *t*(13)= 1.08 | .302 |
| Duration Score | 11.86 (6.2) | 9.86 (6.2) | .74 | .003 | *t*(13)= 1.81 | .093 |
| Parental Emotional Abuse (PEA) | 2.50 (1.7) | 2.71 (1.6) | .88 | <.001 | *t*(13)= -1.00 | .336 |
| Parental Physical Abuse (PPA) | 2.00 (1.7) | 1.93 (1.7) | .93 | <.001 | *t*(13)= 0.43 | .671 |
| Physical and Emotional Abuse by Siblings (PEAS) | 0.50 (1.2) | 0.21 (0.6) | .85 | <.001 | *t*(13)= 1.75 | .104 |
| Emotional Neglect (EN) | 2.43 (1.6) | 1.79 (1.7) | .55 | .042 | *t*(13)= 1.61 | .133 |
| Physical Neglect (PN) | 1.14 (1.3) | 1.00 (1.3) | .82 | <.001 | *t*(13)= 0.81 | .435 |
| Witnessed Violence towards Parents (WITP) | 0.36 (0.6) | 0.71 (1.0) | .83 | <.001 | *t*(13)= -2.11 | .055 |
| Witnessed Violence towards Siblings (WITS) | 1.71 (2.0) | 1.36 (1.8) | .93 | <.001 | *t*(13)= 1.79 | .096 |
| Physical and Emotional Abuse by Peers (PEER) | 1.79 (1.4) | 1.64 (1.5) | .83 | <.001 | *t*(13)= 0.56 | .583 |
| Sexual Abuse by a Member of the Household (SEXA-H) | 0.57 (1.2) | 0.57 (1.2) | 1.00 | n/a | n/a | n/a |
| Sexual Abuse by Others Not Living in the Same Household (SEXA-O) | 1.714 (1.8) | 1.43 (1.3) | .97 | <.001 | *t*(13)= 1.30 | .218 |

*N* = 14. SD = standard deviation.


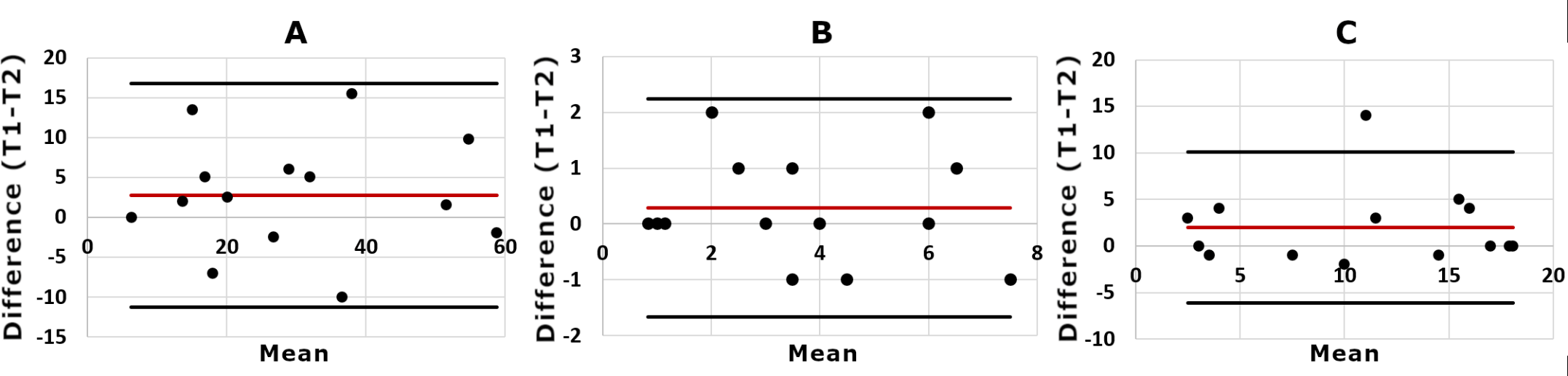
**S3 Figure. Bland and Altman analysis of test-retest reliability for the KERF-40+ global scores (i.e., sum, multiplicity, and duration score).**

In panel A, the plot depicts the sum score, in panel B the multiplicity score, and in panel C the duration score. For each plot, the mean of (x-axis) and difference between (y-axis) T1 and T2 were calculated. The red line indicates the mean difference between test and retest scores. The black horizontal lines indicate the test-retest confidence interval ((mean of the difference between T1 vs. T2) +/- 1.96*(standard deviation of T1 vs. T2)). For illustrative purposes, dots were jittered alongside the x-axis using the normal distribution function in Microsoft Excel.
